# Peripherally Inserted Central Venous Catheter-Related Complications in Adult Patients with Haematological Malignancy

**DOI:** 10.21315/mjms2024.31.2.6

**Published:** 2024-04-23

**Authors:** Kee Wei Lee, Chin Sum Cheong, Gan Gin Gin

**Affiliations:** Department of Medicine, Faculty of Medicine, Universiti Malaya, Kuala Lumpur, Malaysia

**Keywords:** peripherally inserted central venous catheter, complications, haematological malignancies

## Abstract

**Background:**

Peripherally inserted central venous catheters (PICC) are widely used in patients with haematological malignancies owing to the requirement for prolonged intravenous therapy. However, the growing use of PICCs has resulted in a multitude of complications such as infections and thrombosis, leading to prolonged hospitalisation periods and increased morbidity. This study aimed to determine the incidence of and factors associated with PICC-related complications in patients with haematological malignancies.

**Methods:**

This prospective cohort study was conducted at a single academic institution. The inclusion criteria involved all adult patients with haematological malignancies who had newly inserted PICCs. The patients were observed for a minimum duration of 60 days to evaluate the incidence of PICC-related infections and thrombosis, as well as mechanical complications.

**Results:**

A total of 119 PICCs were implanted in 85 patients. Among them, more than half of the patients were diagnosed with lymphoma (55.0%). The median dwell time was 61 days (interquartile range: 98 days). The incidence of PICC-related complications was 58.0% (6.9 per 1,000 catheter-days). Specifically, 43 PICCs (36.1%, 4.3 per 1,000 catheter-days) experienced infective complications, 25 (21.1%, 2.5 per 1,000 catheter-days) encountered mechanical complications and 1 (0.8%, 0.1 per 1,000 catheter-days) exhibited thrombotic complications. Furthermore, an underlying diagnosis of acute leukaemia was significantly associated with a higher incidence of PICC-related infections.

**Conclusion:**

Our study revealed higher incidence rates of PICC-related complications in adult patients with haematological malignancies compared to the finding of other studies. Notably, patients with underlying acute leukaemia displayed a higher incidence of PICC-related infections. These findings underscore the importance of implementing appropriate interventions and conducting thorough root cause analyses to effectively mitigate this complication and improve patient outcomes.

## Introduction

The introduction of central venous catheters (CVC) has revolutionised the modern approach to patient management across multiple disciplines. Given their ability to provide central venous access for both diagnostic and therapeutic interventions, CVC play a crucial role in the management of medically complex and acutely ill patients. Central venous access can be established by utilising either conventional centrally inserted central venous catheters (CICC) or peripherally inserted central venous catheters (PICC).

The utilisation of PICC has become increasingly popular, especially among haemato-oncology patients, owing to its capacity to offer long-lasting venous access for chemotherapy administration and blood withdrawal. Compared to CICC, PICC are associated with a lower risk of complications such as pneumothorax or haemorrhage. Moreover, the less invasive insertion techniques and ease of PICC removal offer additional advantages, facilitating the transition from in-patient to out-patient settings in certain situations (1, 2).

However, it is important to note that the use of PICC can be accompanied by complications, including infections, line thrombosis and device-related issues such as occlusion, dislodgement or leakage (3–5). Catheter-related infections are typically considered to be less prevalent in PICC compared to traditional CVC. Contrary to this perception, a study conducted by Chopra et al. (6) revealed that the incidence of PICC-related bloodstream infections (BSI) was comparable to that of CVC-related BSI. Other complications, such as catheter-related thrombosis and device-related problems, have also been reported to be relatively high, ranging from 2%–25.7% and 4%–34%, respectively (1–5, 7). These complications have consistently been associated with prolonged hospitalisation and increased healthcare costs (8, 9).

Although PICC are now considered essential in the management of patients with haematological malignancies, there is a lack of comprehensive understanding regarding the complications associated with PICC in developing countries, particularly in Southeast Asian regions such as Malaysia. Thus, the primary objective of this study was to determine the incidence rate of PICC-related complications in patients with haematological cancers and identify potential risk factors. It is anticipated that the findings of this study will offer relevant insights that can contribute to the formulation of strategies to minimise complications and enhance patients’ outcomes.

## Methods

This single-centre, prospective cohort study was conducted between January 2019 and November 2020 in the haematology unit of an academic institution in Malaysia.

Patients who underwent PICC insertion during this period were identified in the in-patient ward and out-patient daycare units. Patients above 18 years old of age, diagnosed with haematological malignancies and who underwent PICC insertion at our centre were included. Patients with a pre-existing PICC prior to the recruitment period were excluded. In our centre, the general protocol involved the routine removal of PICCs approximately 6 months after insertion, unless there are specific indications for earlier removal.

Information regarding clinical and sociodemographic characteristics was obtained by conducting interviews with patients and/or caregivers, as well as from patients’ electronic medical records. The collected catheter-related information included indications for PICC insertion, whether the insertion was planned or unplanned, the patient’s previous history of insertions, and the number of attempts made during the insertion procedure. The patients underwent weekly follow-up evaluations for a minimum of 60 days following PICC insertion.

Based on an extensive review of the literature, it was observed that the incidence rate of PICC-related complications varied from 10.3% to 24.7%. Taking this information into account, we estimated the expected complication rate in our study to be 15% (2, 7, 10). Hence, we calculated a sample size of 119 insertions to achieve a 95% confidence level and a 5% precision level.

### Catheter-Related Complications

The catheter-related complications assessed during this period of study included infections, catheter-related thrombosis and device-related mechanical complications such as dislodgement, leakage or occlusion.

PICC-related infections included the following categories:

- Local PICC exit-site infection: characterised by the presence of purulent discharge with local manifestations of infection, such as tenderness, erythema or induration within a 2 cm radius from the PICC exit site, without any systemic signs of sepsis (10).
- PICC-related BSI: characterised by the presence of bacteraemia in a patient with an in-situ PICC, within 48 h prior to the onset of symptoms (fever > 38 °C or hypotension), in the absence of any other identifiable sources of infection, along with isolation of the same micro-organism from both the PICC lumen and peripheral blood cultures (10).

PICC-related venous thrombosis was characterised by the presence of a thrombus detected by ultrasonography or angiography in the presence of an indwelling PICC.

Mechanical complications included the following categories:

- PICC dislodgement: characterised by the complete or partial removal (displacement outwards by more than 4 cm from its original marking position) by patients or healthcare providers accidentally or unintentionally (11).
- PICC leakage: characterised by the leakage of blood or infusate from the exit-site or along the external length of the catheter lumen.
- PICC occlusion: characterised by the irreversible obstruction of the PICC, leading to the inability to infuse or aspirate fluids, even with the application of manual pressure applied on a 10 mL syringe’s piston, which ultimately led to its removal.

### Dwell Time

Dwell time was defined as the duration from PICC insertion to the occurrence of one of the following outcomes:

- Occurrence of PICC-related mechanical complications, thrombosis or local exit-site infections requiring its removal.
- First positive blood cultures identified as central line-associated blood stream infection (CLABSI).
- PICC removal due to completion of therapy or on patient’s request.
- The demise of the patient.

### Statistics

All collected data were analysed using the Statistical Package of Social Science (SPSS) (version 21.0; IBM SPSS statistics). Statistical significance was defined as *P* < 0.05. Descriptive statistics were used to summarise the demographic characteristics of the study population. Continuous data, such as age, were reported as mean (standard deviation [SD]) or median (interquartile range [IQR]) based on the distribution of data, while categorical data, such as sex, ethnicity, diagnosis, catheter data and PICC complications, were reported as frequency and percentage. PICC-related complications were reported as the rate per 1,000 catheter-days (number of PICC complications divided by the total number of catheter-days and multiplied by 1,000 to derive a rate per 1,000 catheter-days). In the analysis, each PICC insertion was considered an individual event; thus, all reported calculations were based on the number of PICC insertions rather than the number of individual patients.

Inferential statistics, such as the Pearson’s chi-square and Fisher’s exact tests, were used to assess any demographic and clinical differences between patients with and without PICC complications.

## Results

A total of 119 PICC insertions in 85 patients were included in this study. The mean age of the patients was 48.33 (SD 13.86) years old. Lymphoma was the most prevalent diagnosis among patients, followed by leukaemia and other haematological malignancies. Twenty-four patients required more than one PICC insertion during the study period: two times in 16 patients, three times in seven patients and five times in one patient. The demographic, clinical and catheter-related information of the patients are shown in Table 1. The inserted PICCs accounted for 9,968 catheter days, with a median dwell time of 61 days (IQR 98 days). PICC-related infections were the most common complications (36.1%), followed by mechanical complications (21.1%) and catheter-related thrombosis (0.8%). Complication rates per catheter day are presented in Table 2.

Forty-three (36.1%) PICC were removed due to infection, while 26 (21.9%) were removed due to mechanical complications. Table 3 shows the analysis of different clinical and catheter-related factors associated with PICC-related complications and infections. After conducting univariate analysis, it was determined that an underlying diagnosis of acute leukaemia had a significantly higher incidence of PICC-related complications and infections, with a *P*-value of 0.016 and 0.014, respectively.

## Discussion

In this observational study, the incidence rate of PICC-related complications was 58.0% (6.9 per 1,000 catheter-days). This finding was higher than those reported in other studies, where the incidence rate ranged from 10.3% to 24.7% (2, 7, 11). PICC-related infections were the most reported complications among our patients. The incidence of PICC-related infections in this study was 36.1% (4.3 per 1,000 catheter-day), with the majority being classified as CLABSI. Comparatively, the incidence of infectious complications at our centre was higher than that reported by other centres (7, 11). Previous studies have highlighted the significant impact of PICC-related infections, particularly CLABSI, which can result in prolonged hospitalisation for intravenous antibiotic treatment and may contribute to worse patient outcomes (12). This is of particularly concerning because PICC-related infections can have a negative impact on a patient’s quality of life and clinical outcomes. Additionally, these infections lead to increased healthcare costs due to prolonged hospitalisation and the need for PICC reinsertion.

In this study, the incidence of PICC-related CLABSI is lower in comparison to the reported rates of CVC-related BSI among patients with haematological malignancies, which range from 5.6 to 17.3 per 1,000 CVC days (13, 14). Although our findings differ from those of a previously conducted meta-analysis, it is noteworthy that the patient cohort included in their analysis was different from our study population (6).

PICC-related infections have been demonstrated to be associated with various factors, including patient-related factors, such as underlying disease and extent of immunosuppression. Additionally, catheter-related factors, such as the number of lumens and types of catheters, as well as the insertion techniques and PICC care, can also contribute to the risk of infection (15, 16). Patients with haematological malignancies are at a higher risk of infection as compared to those with solid tumours owing to the more severe immunosuppressive status, such as prolonged neutropenia, as well as the use of more intense cytotoxic chemotherapy in this group of patients (13, 17). In addition, infusates administered through PICC play an important role in the risk of infective complications. As evidenced in a study by Al-Tawfiq et al. (18), chemotherapeutic infusions were associated with higher infection rates compared to anti-microbial infusions. Thus, it is not surprising that patients with haematological malignancies undergoing therapy are inherently at an increased risk of infection. Among haematological malignancies, acute leukaemia had been shown to be associated with higher infection rates (19). Notably, approximately half of our patients were diagnosed with underlying acute leukaemia and were undergoing treatment. This could be the explanation for the relatively high incidence rate of PICC-related complications in our study.

Device-related factors, such as the use of antibiotic-impregnated PICC and the presence of positive pressure valves, have been shown to be effective in preventing CLABSI (20, 21). However, most PICC used in this institution were non-antibiotic-impregnated, which could be a plausible reason for the high incidence rate of PICC-related infections compared to other centres. Another possible explanation is the significant proportion of patients in our study had prior history of PICC insertions since previous PICC use has been identified as a risk factor for CLABSI (13). However, due to the limited sample size, we were unable to establish statistical significance in our findings.

Certain non-modifiable factors, including climate, can impact infection rates. Research by Gao et al. (7) revealed a significant association between PICC in-dwelling during the summer season with increased incidence of PICC infections compared to other seasons. The hot and humid environment during the summer has been proposed to promote bacterial colonisation and growth. Given that Malaysia is a tropical country, this factor may account for the higher infection rates observed in our local context.

In this study, mechanical complications such as PICC leakage, occlusion and dislodgement, occurred at an incidence rate of 3.4%–10.1% (0.4–1.2 per 1,000 catheter-day), and these findings were consistent with the findings of other studies (7, 22). Only one PICC-related venous thrombosis was reported in this study, which was lower than that reported in other studies (7, 23, 24). The risk factors for catheter-related thrombosis include the number of insertion attempts, catheter size and insertion by non-radiologists. Since all PICC insertions were performed under ultrasound guidance by radiologists and the majority were inserted in one attempt, this may account for the low incidence of PICC-related thrombosis in this study. However, there is also a possibility that this condition may have been underdiagnosed because of the absence of clinical symptoms or signs, as studies have shown that only 4% of confirmed PICC-related venous thromboses are symptomatic (25, 26).

It is widely accepted that programmes, such as catheter care bundles, are crucial for preventing catheter-related complications especially CLABSI (27–30). Most hospitals in Malaysia, including our centre, implemented the programme more than 5 years ago. Utilising maximal barrier precautions and applying skin antiseptics during CVC insertion, coupled with daily assessment of the CVC post-insertion and indications for removal, are crucial strategies to minimise infection risks. However, the incidence of CLABSI in our unit was relatively high necessitating the need for a root cause analysis to address this issue. Recently published recommendations address the pre- and post-insertion practices of catheters, emphasising the use of antimicrobial-impregnated catheters, as an additional approach to reduce the incidence of complications and infections (31). A reassessment of our current practices, including the implementation of new strategies like the utilisation of antimicrobial-impregnated catheters, may be warranted. Although our centre conducts regular surveillance, including monthly audits and monitoring of CLABSI incidence specific to each unit, it is essential to adopt proactive preventive strategies that can significantly lower the incidence of PICC-related complications. Comprehensive education on catheter care should be inclusive of not only physicians and nurses but also patients and their caregivers. This is especially relevant because most of our patients are discharged from the hospital with PICC. Patients with indwelling PICC usually undergo a weekly review consistent with international recommendations (10, 32). Empowering patients or caregivers to conduct daily inspections of the catheter site enables early detection of potential complications. Regular reinforcement of this training, as shown in previous studies, is crucial in reducing infection rates (33).

The primary limitations of this study included its relatively small sample size and single-centre design. Nevertheless, it is worth highlighting that this is the first study, within this region to examine PICC-related complications in patients with haematological cancers.

## Conclusion

The high incidence of PICC-related infections observed in this study warrants further evaluation through larger multicentre studies. Adoption of the strategies outlined in recent guidelines could be advantageous in effectively mitigating these complications.

## Figures and Tables

**Table 1:** Demographic, clinical and catheter characteristics (*n* = 119)

Patient characteristics	*n* (%)
Age (years old)	
Mean	48.33 (13.86)^a^
< 60	95 (79.8)
60	24 (20.2)
Gender	
Male	56 (47.1)
Female	63 (52.9)
Ethnicity	
Malay	61 (51.3)
Chinese	43 (36.1)
Indian	13 (10.9)
Others	2 (1.7)
Diagnosis	
Lymphoma	66 (55.5)
Leukemia	50 (42.0)
Others	3 (2.5)
Setting	
Planned insertion	56 (47.1)
Unplanned (emergency)	63 (52.9)
Previous PICC use	
Yes	57 (47.9)
No	62 (52.1)
Site of PICC insertion (arm)	
Right	86 (72.3)
Left	33 (27.7)
Vein of PICC insertion	
Basilic	75 (63.0)
Cephalic	17 (14.3)
Brachial	26 (21.8)
Others	1 (0.9)
Number of attempts for insertion	
1	107 (89.9)
> 1	12 (10.1)

Notes:

a mean (standard deviation);

PICC = peripherally inserted central venous catheter

**Table 2:** Incidence of PICC-related complications

PICC complications	*n* (%)^a^	Incidence^b^
CLABSI	32 (26.9)	3.2
Local exit-site infection	11 (9.2)	1.1
PICC occlusion	12 (10.1)	1.2
PICC dislodgement	9 (7.6)	0.9
PICC leakage	4 (3.4)	0.4
PICC-related venous thrombosis	1 (0.8)	0.1

Notes:

a based on a total of 119 PICC insertions;

b based on total number of catheter days of 9,968;

PICC = peripherally inserted central venous catheter

**Table 3:** Factors associated with PICC-related complications and PICC-related infections

	PICC related complications			PICC related infections		

Clinical characteristics	With complication *n* = 69 (%)	Without complication *n* = 50 (%)	*P-*value	With infection *n* = 43 (%)	Without infection *n* = 76 (%)	*P-*value
Age range			0.672			0.426
< 60	56 (58.9)	39 (41.1)		36 (37.9)	59 (62.1)	
60	13 (54.2)	11 (45.8)		7 (29.2)	17 (70.8)	

Gender			0.569			0.500
Male	34 (60.7)	22 (39.3)		22 (39.3)	34 (60.7)	
Female	35 (55.6)	28 (44.4)		21 (33.3)	42 (66.7)	

Ethnicity			0.153^a^			0.650^a^
Malay	38 (62.3)	23 (37.7)		23 (37.7)	38 (62.3)	
Chinese	20 (46.5)	23 (53.5)		14 (32.6)	29 (67.4)	
Indian	10 (76.9)	3 (23.1)		6 (46.2)	7 (53.8)	
Others	1 (50.0)	1 (50.0)		0 (0.0)	2 (100.0)	

Diagnosis			0.016*^a^			0.014*^a^
Lymphoma	32 (48.5)	34 (51.5)		18 (27.3)	48 (72.7)	
Leukemia	36 (72.0)	14 (28.0)		25 (50.0)	25 (50.0)	
Others	1 (33.3)	2 (66.7)		0 (0.0)	3 (100.0)	

Setting			0.569			0.291
Planned	34 (60.7)	22 (39.3)		23 (41.1)	33 (58.9)	
Unplanned	35 (55.6)	28 (44.4)		20 (31.7)	43 (68.3)	

Previous PICC			0.469			0.820
Yes	35 (61.4)	22 (38.6)		20 (35.1)	37 (64.9)	
No	34 (54.8)	28 (45.2)		23 (37.1)	39 (62.9)	

Arm of insertion			0.638			0.694
Right	51 (59.3)	35 (40.7)		32 (37.2)	54 (62.8)	
Left	18 (54.5)	15 (45.5)		11 (33.3)	22 (66.7)	

Vein of insertion			0.938^a^			0.906^a^
Basilic	42 (56.0)	33 (44.0)		28 (37.3)	47 (62.7)	
Cephalic	10 (58.8)	7 (41.2)		5 (29.4)	12 (70.6)	
Brachial	16 (61.5)	10 (38.5)		10 (38.5)	16 (61.5)	
Others	1 (100.0)	0 (0.0)		0 (0)	1 (100.0)	

Insertion attempt			0.061			0.831
1	59 (55.1)	48 (44.9)		39 (36.4)	68 (63.6)	
> 1	10 (83.3)	2 (16.7)		4 (33.3)	8 (66.7)	

Median time, day (IQR)	Median	Median 116 (99.3)	< 0.01*	Median 33 (52)	Median	0.003*
	38 (54)				88 (108)	

Notes:

a using Fisher’s exact test;

* *P* < 0.05 is statistically significant;

PICC = peripherally inserted central venous catheter; IQR = interquartile range
